# Hymenoptera venom allergy in children

**DOI:** 10.1186/s13052-024-01731-9

**Published:** 2024-12-20

**Authors:** Mattia Giovannini, Francesca Mori, Simona Barni, Francesca Saretta, Stefania Arasi, Riccardo Castagnoli, Lucia Liotti, Carla Mastrorilli, Luca Pecoraro, Lucia Caminiti, Gunter Johannes Sturm, Gian Luigi Marseglia, Michele Miraglia del Giudice, Elio Novembre

**Affiliations:** 1 Allergy Unit, Meyer Children’s Hospital IRCCS, Florence, 50139 Italy; 2 Department of Health Sciences, University of Florence, Florence, 50139 Italy; 3 Pediatric Department, Latisana-Palmanova Hospital, Azienda Sanitaria Universitaria Friuli Centrale, Udine, 33100 Italy; 4 Allergy Diseases Research Area, Pediatric Allergology Unit, Bambino Gesù Children’s Hospital IRCCS, Rome, 00165 Italy; 5 Department of Clinical, Surgical, Diagnostic and Pediatric Sciences, University of Pavia, Pavia, 27100 Italy; 6 Pediatric Clinic, Fondazione IRCCS Policlinico San Matteo, Pavia, 27100 Italy; 7 Pediatric Unit, Department of Mother and Child Health, Salesi Children’s Hospital, Ancona, 60123 Italy; 8 Pediatric Hospital Giovanni XXIII, Pediatric and Emergency Department, AOU Policlinic of Bari, Bari, 70126 Italy; 9 Pediatric Unit, Department of Surgical Sciences, Dentistry, Gynecology and Pediatrics, University of Verona, Verona, 37126 Italy; 10 Department of Human Pathology in Adult and Development Age “Gaetano Barresi”, Allergy Unit, Department of Pediatrics, AOU Policlinico Gaetano Martino, Messina, 98124 Italy; 11 Department of Dermatology and Venerology, Medical University of Graz, Graz, Austria; 12 Allergy Outpatient Clinic Reumannplatz, Vienna, Austria; 13 Department of Woman, Child and General and Specialized Surgery, University of Campania Luigi Vanvitelli, Naples, 80138 Italy

**Keywords:** Hymenoptera, Venom, Allergy, Children, Pediatrics

## Abstract

From a taxonomic point of view, Hymenoptera are subclassified into families: *Apidae*, including honeybees (*Apis mellifera*) and bumblebees (Bombus), and Vespidae, which, in turn, are divided into the subfamilies of Vespinae (wasps, including hornets, vespules, dolichovespules) and Polistinae (paper wasp). Hypersensitivity to Hymenoptera venom can be linked to immunological (IgE-mediated or non-IgE-mediated) and non-immunological mechanisms. Reactions are classified into local reactions, large local reactions, systemic reactions, toxic reactions, and unusual reactions. In general, children sensitize less frequently and have less severe reactions than adults, probably due to less exposure to repeated stings and fewer comorbidities. There are risk factors for systemic reactions that should be discussed with patients and their parents as appropriate. A correct diagnosis of Hymenoptera venom allergy relies on a careful clinical history and the appropriate use of skin and in vitro tests. The in vitro tests include serum specific IgE toward venom extracts and toward allergenic molecules. In complex diagnoses, CAP-inhibition and the Basophil Activation Test can also be used. In the presence of a systemic reaction, the basal serum tryptase measurement should be performed to rule out mastocytosis. In case of allergic reactions to Hymenoptera stings, in the acute phase, according to the current guidelines, the treatment of signs and symptoms mainly includes the use of adrenaline as first-line treatment in case of anaphylaxis and antihistamines and corticosteroids as subsequent lines of treatment. Given the impossibility of avoiding a new sting with certainty, the treatment of choice in subjects with hypersensitivity to Hymenoptera venom who have experienced systemic reactions is based on venom immunotherapy (VIT), with the venom of the responsible stinging insect identified after an adequate allergological work-up. VIT is performed in a suitable environment and has proved to be safe and effective with various administration protocols, both accelerated and conventional. The prevention of Hymenoptera venom anaphylaxis in patients who have already developed a previous episode is crucial and must be supported by environmental protection interventions and early therapy. Places where one is more likely to encounter insects and risky behaviors should be avoided.

## Stinging Hymenoptera and their allergens

Hymenoptera venom allergy represents an important cause of morbidity and mortality both in adulthood and pediatric age [1]. From a taxonomic point of view, Hymenoptera are subclassified into families: *Apidae*, including honeybees (*Apis mellifera*) and bumblebees (Bombus), and *Vespidae*, which, in turn, are divided into the subfamilies of *Vespinae* (wasps, including hornets, vespules, dolichovespules) and *Polistinae* (paper wasp) [2]. Finally, ants are also part of the order Hymenoptera [3].

Hymenoptera venom allergy is caused by the allergens contained in Hymenoptera venom. Generally, it contains, e.g., histamine, serotonin, tyramine, catecholamines, mastoparans, kinins, chemoattractant peptides, phospholipases, hyaluronidase, melittin, and antigen 5 [4]. Among these components, phospholipases A1 and A2 seem to represent the allergens most involved in triggering allergic reactions [5]. Moreover, these allergens are linked to an increased risk of anaphylaxis [5]. Hymenoptera generally sting humans for defense or to protect their nests and hives from predators [1].

The honeybee is the only type of Hymenoptera that leaves both the serrated stinger and the poison sac into the skin of the stung subject, causing evisceration and death [6]. On the other hand, the yellow jacket has a smooth stinger and can cause multiple stings [4]. The amount of venom released during a sting varies in species of Hymenoptera and even within the same species (Table 1) [6–8].

**Table 1:** Characteristics of the main Hymenoptera and quantity of venom released during a sting; modified from [1]

**Hymenoptera**	**Venom/sting**	**Characteristics**
**Honeybees**	50–140 mcg	Stocky body covered with hair; 1–1.5 cm; serrated sting; herbivore; hive defense
**Bumblebees**	10–31 mcg	Massive body, hairy, black with streaks, white or reddish spot at the end of the abdomen; 2–3 cm; smooth sting; non-aggressive; used for pollination of plants in greenhouses (they do not need to see the sun for orientation)
**Yellow jackets**	1.7–3.1 mcg	Truncated abdomen (crossed shield), yellow with black streaks; 1.5–2 cm; smooth sting; aggressive, carnivorous: insect eater; attracted by food: meat, fish and sugary substances
**Hornets**	Venom dry weight poison sac 260 mcg	Massive yellow, black and rusty body; 2.5–3.5 cm; smooth sting; moderate aggression, night flyer
**Paper wasps**	4.2–17 mcg	Tapered body with ogival abdomen, black with yellow stripes, thin legs; 1–1.7 cm; moderate aggression

Specifically, honeybees, bumblebees, yellow jackets, hornets, and paper wasps release a different quantity of venom [6–8]. It is not possible to rigorously estimate the exact amount of venom released in each sting through laboratory methods [7]. Moreover, the volume of injected venom is variable [7].

When someone has been stung by a Hymenoptera species, it is not always possible to visualize the type of Hymenoptera involved [1]. At the same time, it is important to recognize it to carry out a correct diagnostic therapeutic procedure [1]. Specifically, it is recognized in 73% of cases through the use of an entomological notice board during a visit to the allergist [1]. In other cases, its recognition can be facilitated through the knowledge of specific peculiarities of each Hymenoptera, such as the aspect and positioning of the nest [1]. In particular, *Vespa*,* Vespula*, and *Dolichovespula* generally make up their nests in an underground environment. Hornets create nests inside of hollow trees. Finally, *Polistes* nests are often under roof coverings [1].

Moreover, it is important to determine the area where the Hymenoptera sting occurred [1]. Specifically, the rate of primary sensitization to Hymenoptera venom is higher in rural areas than in urban ones [9]. Finally, some subjects have a higher rate of Hymenoptera stings than the general population. It was demonstrated that beekeepers’ children, relatives, or neighbors are 2–3 times more at risk of being stung by a Hymenoptera [10]. Additionally, the consequences of the stings of different species of Hymenoptera are not comparable. Specifically, epidemiological studies report a comparable risk of anaphylaxis as a result of honeybee and wasp stings and, at the same time, a 2.74-fold increased risk as a result of hornet stings [11]. The knowledge of the peculiarities of each species of Hymenoptera is of fundamental importance to allow the allergist to carry out a diagnostic therapeutic procedure for each patient.

## Epidemiology

Hymenoptera venom sensitization rates are 3.7% of children in an Italian study [12]. The prevalence of large local reactions (LLRs) ranges between 0.9% in Italy and 11.5% in Israel, while the prevalence of systemic reactions (SRs) is reported to be less than 1% in an Italian study, resulting, however, higher in an Israeli study (6.5%) [13, 14]. An Israeli study, based on the use of questionnaires, has reported a percentage of moderate-severe SR equal to 2.5% of cases [14]. Data relating to the epidemiology of reactions to Hymenoptera venom in children are shown in Table 2.

**Table 2:** Pediatric studies on the epidemiology of reactions to Hymenoptera stings and possible association with atopic predisposition. Data expressed as absolute number of patients, percentages in brackets

**Authors, Year**	**Country**	**Number of patients**	**Hymenoptera sting history**	**Prevalence LLR**	**Prevalence MSR**	**Prevalence SSR**	**Atopy**
Novembre et al., 1998 (questionnaire + SPT) [12]	Italy (Florence)	1175	228 (19.4%)	224 (19.06%)	4 (0.34%)		Related
Graif et al., 2006 [14] (questionnaire)	Israel (Tel Aviv)	10,021	5624 (56.3%)	1156 (11.5%)	654 (6.5%)	250 (2.5%)	Related
Jennings et al., 2010 (questionnaire) [15]	Ireland (Cork)	4112	1544 (37.5%)	90 (2.2%)	53 (1.3%)	8 (0.2%)	Related
Quercia et al., 2014 [13] (questionnaire)	Italy (Ravenna)	1035	173 (16.7%)	9 (0.9%)	5 (0.5%)	1 (0.01%)	

*LLR* Large local reactions, *MSR* Mild systemic reactions, *SPT* Skin prick test, *SSR* Moderate-severe systemic sting reactions

Systemic anaphylactic reactions related to Hymenoptera venom represents 20% of the total anaphylaxis in the pediatric population [16]. Fatal cases in children are being reported from 5 years of age [17].

## Clinical presentation and pathogenesis

Hypersensitivity to Hymenoptera venom can be linked to immunological (IgE-mediated or non-IgE-mediated) and non-immunological mechanisms. Reactions are classified into local reactions, LLRs, SRs, toxic reactions, and unusual reactions.

### Local reactions

Generally, insect stings in non-allergic subjects cause a local reaction characterized by, e.g., pain, erythema, and edema in the region of the puncture. Lesions resolve within 24 h, sometimes hesitating in a small peri-lesional reaction visible for a few days.

### LLRs

In LLRs, edema starts from the puncture site, spreads with a diameter greater than 10 cm and lasts more than 24 h. Other clinical manifestations, such as asthenia, fever, headache, and loco-regional lymphadenopathies and/or lymphangitis, occur very often. Skin prick tests (SPTs) and/or serum specific IgE (sIgE) for Hymenoptera venom result positive in 70–90% and in 26–50% of cases, respectively. Mostly, an IgE-mediated reaction has been hypothesized, while in other cases, a cell-mediated response or a combination of both has been suspected based on clinical aspects and in vivo and in vitro examinations [4].

### SRs

SRs (anaphylaxis or non-anaphylaxis), mostly IgE-mediated reactions involving, e.g., skin and mucous membranes, respiratory system, cardiovascular system, gastrointestinal system, and nervous system, may also be determined by different immunological mechanisms, such as the activation of complement mediated by IgG-venom complexes [4]. Symptoms and signs often appear within a few minutes or a few hours from the puncture. Currently, from a diagnostic point of view, anaphylaxis from Hymenoptera venom, as well as from different etiologies, follows the criteria dictated by the guidelines, while to define the degree of severity of SRs, different classifications have been proposed over time [18].

The classifications of Muller [19] and Ring and Messmer [20] are currently the most used, but they show several limitations; the first classification does not take into account the possible absence of skin manifestations and the possibility that an isolated cardiovascular shock may be the only systemic manifestation, while in the second classification, the cardiovascular involvement is considered much more severe than the respiratory one. The European Academy of Allergy and Clinical Immunology proposed the current classification of acute allergic reactions, dividing them into local and SRs and attributing the degree of severity according to symptoms/signs reported [21]. In children, over 60% of SRs described are mild and limited to the skin, while in adults, respiratory or cardiovascular manifestations occur in 70% of cases [22].

### Toxic reactions

TRs are caused by some components of the venom (e.g., phospholipase, hyaluronidase), are dose-dependent, and take place in cases of multiple simultaneous punctures. Frequent clinical manifestations are, e.g., nausea, vomiting, diarrhea, headache, dizziness, and fever. Cardiac complications, intravascular hemolysis including coagulation disorders with bleeding, renal insufficiency, and rhabdomyolysis are possible. Clinical manifestations occur within a few hours or days, and their severity depends on the number of stings. Mortality can be related either to hypersensitivity reactions (not dose-dependent and not related to the number of bites) or to the massive release of venom [23–25].

### Unusual reactions

Unusual reactions represent the rarest reactions, and the relation to the sting of Hymenoptera sometimes appears dubious and based on case reports [23].

### Risk factors for SRs

Despite the possibility of fatal anaphylaxis the first time a child encounters Hymenoptera venom [1], there is no certain evidence regarding risk factors linked to the onset of severe SRs in children. Nonetheless, even when the data come from large study populations [15, 26–29], self-administered or parent-administered questionnaires are often the only tool used to assess the severity of reactions, with the risk of overestimation related mainly to the lowest alarm threshold of atopic patients to adverse reactions.

The main risk factors for serious reactions reported for both children and adults include the following: history of previous Hymenoptera anaphylactic reaction, old age, chronic cardiovascular diseases, lung diseases (including bronchial asthma), drug use as beta-blockers or ACE-inhibitors, type of insect (in the Mediterranean area, the risk of potentially fatal reactions is about three times higher for the hornet compared to other vespids and the honeybee), site of the sting (head and neck stings are associated with an increased risk of fatal reactions), and elevated baseline tryptasemia levels [30]. Some studies [31, 32] have reported a significant association between the presence of atopy, especially asthma, and severe systemic reactions.

**Fig. 1:**
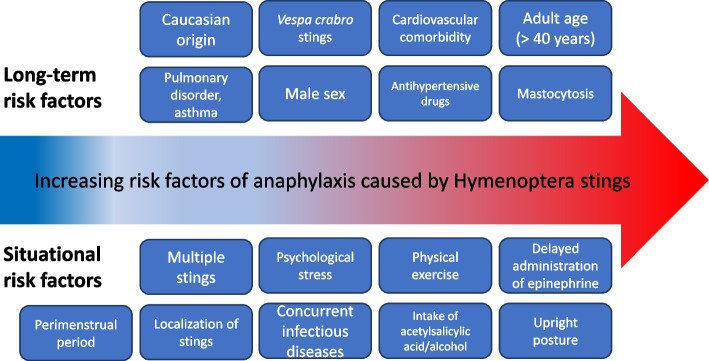
Risk factors of systemic side effects caused by Hymenoptera stings; modified from [32]

A review [32] categorized the risk factors for anaphylactic reactions triggered by Hymenoptera stings into two main groups: situational risk factors and long-term risk factors (Fig. 1).

Among the former, e.g., the delayed administration of epinephrine, upright posture during anaphylaxis, and physical exercise (especially if intense) during or especially after the insect bite have been confirmed as risk factors for a fatal outcome, while there are insufficient data in favor of the localization of the puncture (as recently confirmed [33]), the intake of alcohol or acetylsalicylic acid, concomitant infections, stress, and peri-menstrual status. In the group of long-term risk factors, there is evidence regarding, e.g., the association between systemic mastocytosis and severe anaphylactic reactions. Other relevant risk factors are the male gender as well as cardiovascular comorbidities. Children are at less risk for severe SRs than older patients (> 40 years), who have both a higher incidence of comorbidities and, overall, a greater risk of SRs. Finally, retrospective studies and case series [33–35] seem to question the harmful effect of antihypertensive drugs (i.e., beta-blockers or ACE inhibitors) in the course of anaphylaxis induced by Hymenoptera venom.

Children become sensitized less frequently and have less severe reactions than adults, probably due to less exposure to repeated stings and fewer comorbidities. However, the number of pediatric patients with systemic reactions is significant and deserves a shared management pathway defined at a territorial level. Furthermore, risk factors for severe SRs need to be properly discussed with patients and their parents.

## Diagnosis

Diagnosis is based on the classification of the type of reaction, confirmation of the pathogenesis, and identification of the stinging insect. An accurate medical history is preliminary to a proper diagnosis. The key questions for the clinical history are: Has the insect been recognized? Did the sting remain stuck in the skin (or mucous membrane)? Where did the sting take place? How many stings did the patient receive? What activity was the patient doing at the time of the puncture? Has the patient been stung on other occasions, and if so, with what results? How soon did the clinical manifestations appear? Has the patient taken any drug, and if so, with what outcome? The possible recognition of the insect through the entomological notice board is also crucial [4, 36].

In the case of only local reactions, the diagnosis is essentially clinical, and the execution of allergological tests is optional [1]. Conversely, in the case of suspected SRs, allergological investigations are recommended. They include skin tests (i.e., SPTs and intradermal tests) and/or the search for sIgE for the identification of the Hymenoptera species involved. In any case, these tests should be performed 2 to 4 weeks after the acute adverse reaction in order to avoid false negatives. In fact, the massive release of mediators of the allergic response that occurs in the acute reaction determines a so-called “refractory” period following the reaction itself [4].

### Skin tests

The guidelines suggest performing SPTs as the first diagnostic step. Intradermal tests are indicated as a second diagnostic level only in case of negative skin prick tests. All tests must be performed by expert personnel and in an environment equipped for any reactions [4, 36].

A SPT is performed at a concentration of 100 μg/ml. The intradermal reactions must always start from very low concentrations, according to the signs and symptoms presented by the patient; concentrations from 0.001 up to a maximum of 1 μg/ml are normally used [4]. However, since the child has less skin sensitivity, it has been suggested to start with higher concentrations (0.1 mg/ml) in order to speed up the procedure [37].

The intradermal test is performed by injecting 0.02 ml of the allergenic extract into the dermis, generating a wheal of about 3 mm in diameter. The reading should be performed after 15–20 min. The test is considered positive if there is an increase of at least 3 mm in the mean diameter of the initial wheal or if the diameter of the wheal obtained is double the diameter of the initial wheal [36, 38]. Intradermal tests are indicated even in the event of a positive SPT for the exact identification of the cutaneous endpoint, which is very useful in the follow-up and, in particular, for the evaluation of the efficacy of any specific immunotherapy (venom immunotherapy, VIT) [1, 39].

### sIgE

The measurement of serum venom-sIgE can be performed with various diagnostic techniques (usually immunoenzymatic). The sensitivity of serological tests using whole extracts is generally lower than that of skin tests. In vitro tests for detecting sIgE against the whole venom extract can be negative in some patients with positive skin tests; conversely, some patients with negative skin tests have a positive in vitro test. Therefore, guidelines suggest carrying out both tests [4, 36].

The severity of the reaction does not relate to the result of either in vivo or in vitro diagnostic tests. The greatest positivity of the tests has been found in patients with LLRs, while about 25% of those who have presented a systemic reaction have negative SPT or positive intradermal tests only at a concentration of 1 μg/ml [4]. In some cases of fatal reactions due to Hymenoptera sting, the presence of sIgE may not be identified [40]; however, in these cases, the possibility of mast cell diseases (in which skin tests and sIgE are often negative) has not been evaluated [41].

sIgE toward molecular allergens of the Hymenoptera venom are available and useful, especially in complex diagnoses [42]. The measurement of total IgE can help in the correct interpretation of the result of sIgE, especially in cases of very low levels [42].

### Complex diagnoses

**Fig. 2:**
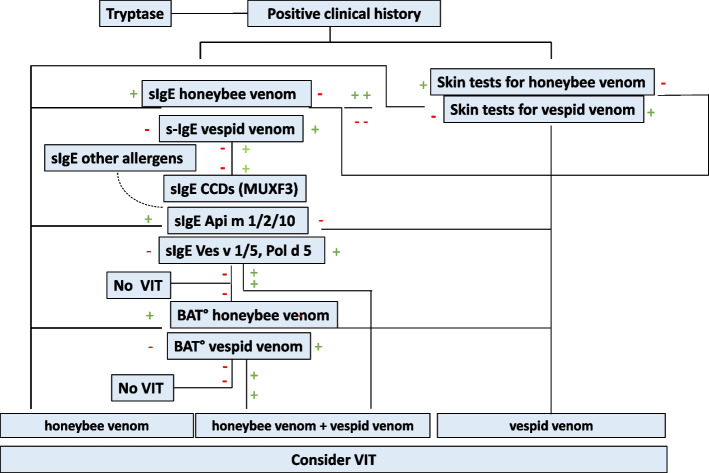
Diagnostic algorithm in honeybee and vespid venom allergy; modified from [42]. ° Not available for routine diagnosis in every clinic; BAT: basophil activation tests, CCDs: cross-reactive carbohydrate determinants, VIT: venom immunotherapy; a red minus indicates a negative, a green plus a positive test result

**Fig. 3:**
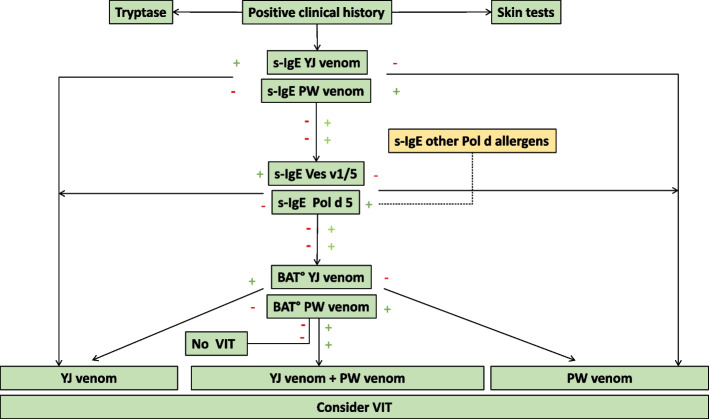
Diagnostic algorithm in yellow jacket and paper wasp venom allergy; modified from [42]. ° Not available for routine diagnosis in every clinic; BAT: basophil activation tests, PW: paper wasp, VIT: venom immunotherapy YJ: yellow jacket; a red minus indicates a negative, a green plus a positive test result

In subjects with a history suggestive of clinically manifest IgE-mediated allergy, in which allergy tests are positive for both *Vespidae* and *Apidae* venom or for both *Vespidae* and *Polistinae* venom, in addition to a careful clinical history aimed at identifying which insect is most likely to have caused the reaction, the search for species-specific molecular allergens (*Apidae*: Api m 1, Api m 2, Api m 3, Api m 5, Api m 10; *Vespidae*: Ves v 1, Ves v 5; *Polistinae*: Pol d 5) may be very useful. The detection of sIgE to cross-reactive molecular allergens (e.g., Cross-Reactive Carbohydrate Determinants, CDDs) may help to discern genuine sensitization from co-sensitization cases [42, 43]. Therefore, molecular allergology may support the allergy specialist in choosing the most suitable venom for VIT and, therefore, avoiding the inappropriate treatment with double venom in some cases [44] (Figs. 2 and 3).

The Basophil Activation Test (BAT) [1] is recommended in cases with a suggestive clinical history and both negative skin tests and sIgE and in cases of double positivity and inconclusive results with molecular allergens tests [1, 45]. The role of BAT as a diagnostic tool in patients with mast cell disorders and negative sIgE and skin tests is controversial [32, 46]. The CAP inhibition [47, 48] evaluates how many sIgE initially dosed as binding a certain venom bind to another venom with which the patient’s serum is previously incubated. However, BAT and CAP inhibition test can only be performed in a few highly qualified centers and has some limitations, such as costs and results that are sometimes difficult to interpret.

In the presence of a SR, basal serum basal tryptase should be dosed, as adults with mast cell disease and/or elevated baseline tryptase levels are at significantly greater risk of developing severe Hymenoptera sting reactions [35, 49]. According to a study, the basal tryptase cut-off that predicts a greater risk of severe reactions to Hymenoptera venom in children is 4.8 mcg/L [50].

Systemic manifestations of mast cell activation in the absence of skin manifestations should lead to the suspicion of mastocytosis. Specifically, the REMA score represents a simple, economical, and reliable tool for establishing which patients deserve further investigation [51]. It is based on demographics (male gender), symptoms and signs observed during acute episodes (absence or presence of urticaria, pruritus, and/or angioedema, presence of presyncope and/or syncope), and basal tryptase levels. A score ≥ 2 has a high predictive value for clonal diseases.

Sting challenge with a live insect is no longer recommended for the diagnosis of adverse reactions to venom, as it is unethical and potentially risky and, in any case, has a low negative predictive value [52].

## Management

In case of allergic reactions to Hymenoptera stings, in the acute phase, according to the current guidelines, the treatment of signs and symptoms mainly includes the use of adrenaline as first-line treatment in case of anaphylaxis and, e.g., antihistamines and corticosteroids as subsequent lines of treatment [53]. In particular, at the time of emergency care discharge, children with anaphylaxis to Hymenoptera venom should receive precise information regarding the management and recognition of future allergic reactions (Table 3).

**Table 3:** Information that should be provided before emergency care discharge for severe reactions to Hymenoptera stings

**Information/action**	**Expected response**
**Description of signs and symptoms of anaphylaxis**	Prompt recognition of a future anaphylactic event
**Information about the risk of biphasic anaphylaxis**	Suspect a subsequent severe event after complete recovery of clinical manifestations and correct management of first reaction
**Training on use of adrenaline autoinjectors**	Correct adrenaline administration using autoinjectors
**Individualized avoidance measures**	Prevent future stings considering factors such as patient’s age, activity, occupation, hobbies, residential conditions, access to medical care and personal anxiety level
**Referral to a specialist**	Correct diagnosis, management (personalized action plan) and follow-up
**Contact for educational programs or patients’ associations**	Training of caregivers (e.g., school staff)

Hymenoptera sting is a potentially life-threatening event, so after emergency treatment for suspected anaphylaxis, patients should be referred to specialists (possibly age-appropriate) with the skills and competencies necessary to accurately investigate, diagnose, and monitor Hymenoptera venom allergy. Referral to an allergist-immunologist is also appropriate for those patients who may require VIT. Otherwise, patients with a negative clinical history should not be investigated because a sensitization to the venoms may be found without a clinical meaning.

In patients with a history of LLR, SPTs and intradermal tests, as well as in vitro tests such as sIgE determination, may be considered optional, at the discretion of the clinician in specific cases, like in patients at a higher risk of a re-sting with recurrent and sizable LLRs, who may benefit from immunotherapy [1]. On this topic, the American College of Allergy, Asthma and Immunology stated that neither diagnostic tests nor adrenaline prescription is necessary in case of LLR (except for those with risk factors or highly exposed to re-stings) [54]. In particular, a study demonstrated that the risk for systemic reactions seems to be low after two LLRs [55]. So far, an agreement has not been reached on the management of LLRs, and allergy investigations and adrenaline prescriptions are still based on the specialist evaluation of the risks and benefits after discussing them with the patient and their family in each case.

Another clinical scenario possibly occurring after a Hymenoptera sting is the development of only cutaneous SRs (mainly urticaria) without any other clinical manifestation involved. In those cases, some particular situations should be deeply analyzed, taking into account, e.g., distance from emergency care, being children of beekeepers, school staff not trained to manage severe allergic reactions, cardiovascular or respiratory conditions, and effects on quality of life. These factors may advise adrenaline prescription [56].

Except for LLRs (especially after a single episode) and cases of only cutaneous systemic involvement where no consensus has been reached on always providing adrenaline autoinjectors, adrenaline should be prescribed to all children with a clinical history of anaphylaxis [22]. Adrenaline autoinjectors should also be provided to children with an elevated baseline serum level of tryptase or mast cell disorders and SRs to Hymenoptera venom, even if treated with VIT [53]. In this context, in children undergoing VIT, adrenaline autoinjectors should be prescribed particularly for those with risk factors for incomplete clinical protection (e.g., very severe onset reaction, adverse reactions during immunotherapy, lack of sting protection during VIT, severe honeybee allergy) (Table 4).

**Table 4:** Indications to prescription of adrenaline autoinjectors^b^; modified from [1]

Children with previous systemic reaction or with large local reaction and high risk of re-stings (e.g., children of beekeepers), before immunotherapy
Children with high tryptase levels and a history of systemic Hymenoptera reactions, regardless of immunotherapy
Children undergoing immunotherapy who, in the maintenance or discontinuation phase, are still at risk of incomplete clinical protection (e.g., very serious reactions at onset, adverse reactions during immunotherapy, lack of protection demonstrated by new Hymenoptera stings during immunotherapy, severe honeybee allergy)
Patients with a history of large local reaction at risk of multiple stings (e.g., children of beekeepers)
Patients with a history of only one large local reaction, as the risk of more severe reactions in the event of a new sting cannot be excluded^a^

^a^In the event of repeated, large local reactions, however, prescribing an adrenaline autoinjector is not necessary, as the risk of systemic reactions is very low

^b^Among patients to be prescribed two adrenaline auto-injectors, consider, e.g., uncontrolled asthmatics (asthma being a risk factor for fatal anaphylaxis), obese patients (i.e., risk of underdosing), patients living far from a hospital, patients with mastocytosis, patients with history of severe systemic reactions who have required multiple doses of adrenaline

Recently, the European Medicines Agency (EMA) has suggested the prescription of two adrenaline autoinjectors, as well as several additional measures, for all patients at risk for anaphylaxis. After the evaluation of all available data, the EMA confirmed that intramuscular administration is the most indicated route for obtaining a rapid response in the treatment of anaphylaxis [57, 58]. The EMA observed that correct administration of adrenaline by autoinjectors is affected by several factors such as needle length, thickness of subcutaneous fat, mode of operation of the autoinjector (whether spring-loaded and/or cartridge-based), angle of placement into the skin, force used to activate it, and the patient’s ability to follow the instructions properly. For all these reasons, healthcare professionals are recommended to advise patients and carers to carry with them autoinjectors at all times and to instruct the patient and carers on how to use them through effective educational material and practical training to ensure their correct use.

**Fig. 4:**
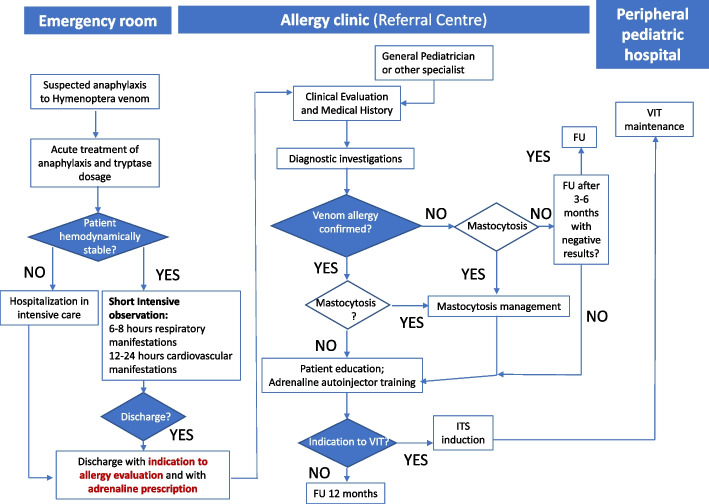
Flow chart process to manage children with Hymenoptera venom allergy; modified from [60]. FU: follow-up, VIT: venom immunotherapy

The management of children with Hymenoptera venom allergy requires an interplay between several specialists: the pediatrician, the allergist-immunologist, and the emergency care physician. All those figures should be trained to give at the beginning information regarding acute management, prevention, and recognition of future reactions. Hymenoptera venom allergy is an underestimated condition representing an important cause of morbidity and mortality worldwide. Few specialized centers are available to manage children with this medical issue who may benefit from VIT. Hence, it is of paramount importance to know where these centers are, how to have access to them, and how to manage children during follow-up [59]. For this reason, we proposed an adapted process to manage children with Hymenoptera venom reactions from the diagram flow for the management of Hymenoptera venom allergy proposed by other *authors* (Fig. 4).

## VIT

Given the impossibility of avoiding a new sting with certainty, the treatment of choice in subjects with hypersensitivity to Hymenoptera venom who have experienced SRs is based on VIT, with the venom of the responsible stinging insect identified after an adequate allergological work-up [61–63].

This type of therapy, including an induction phase and a maintenance phase, is performed by subcutaneous injection in the arm between the elbow and the shoulder for a total duration of 3–5 years. VIT consists of administration in increasing doses, with the starting dose between 0.001 mcg and 0.1 mcg of the selected venom up to the maximum dose of 100 mcg, recommended for the VIT of all Hymenoptera. The maintenance dose should then be given every 4 weeks in the first year, gradually increasing the interval to 6–8 weeks in subsequent years. In specific cases, e.g., a higher maintenance dose (200 mcg) may be indicated in subjects presenting with systemic re-sting reactions during VIT and in the presence of risk factors for multiple stings, as in the case of beekeepers’ offspring [64]. In some pediatric studies even a lower maintenance dose (50 mcg) seems effective [65, 66]. There are different types of protocols for VIT available [67], the choice of which is linked to both organizational and clinical factors (e.g., type of allergen used and severity of previous reactions) [68].

VIT has a specific immuno-modulating power toward Hymenoptera venom and favors the establishment of a state of tolerance toward it. The molecular mechanisms underlying this action have been widely described and act both early and late. Numerous studies have already demonstrated significant variations at the level of innate and adaptive immunity that characterize the response to this treatment: decrease of sIgE and increase of sIgG1/IgG4, reduction of mast cell and basophil activation, specific induction of regulatory T cells and IL-4/IL-5 reduction [69–72]. The indications for carrying out VIT are shown in Table 5.

**Table 5:** Indications for VIT; modified from [1]

Systemic reaction involving other systems than skin with documented sensitization through positive allergy tests (skin tests and/or serum specific IgE)
Systemic skin reaction in the presence of risk factors^a^ with documented sensitization through positive allergy tests (skin tests and/or serum specific IgE)
Systemic reaction in patients with clonal mast cell disease with documented sensitization by positive allergy tests (skin tests and/or serum specific IgE)

^a^E.g., risk of exposure (increased sting risk, as in children of beekeepers), distance from a first aid point, compromised quality of life (e.g., excessive anxiety in the child or their parents)

SRs extending beyond the exclusively cutaneous represent the main indication for performing VIT in the pediatric age. Patients with SRs extending exclusively to the skin appear not to be subject to the future development of more important reactions [63] and are candidates for VIT only in the presence of risk factors. Extended local reactions do not normally represent an indication for performing VIT, even if the latter can be taken into consideration in the case of recurrent and severe LLRs [1]. On the other hand, the random finding of sensitization to Hymenoptera venom is not an indication for VIT (e.g., following allergy multiplex tests) in patients with no clinical history of SRs [64].

As reported in the guidelines [64], children under the age of 5 are rarely subject to SRs following a Hymenoptera sting. In this case, however, the start of VIT can be agreed upon in relation, e.g., to the clinical manifestations and the risk factors of future SRs. In general VIT represents an effective treatment for reducing the risk of a new SR compared to untreated patients [64, 73]. Its effectiveness varies from 77%–84% in the case of honeybee allergy and 91%–96% in the case of wasp allergy [74–78]. At the same time, VIT has proven to be safe, considering that most local adverse reactions occur at the injection site. The latest published pediatric studies also report a low rate of LLRs and a contained rate (4–6% of treated) of SRs, the latter being more frequent in the build-up phase with honeybee venom [79–81]. LLRs are sometimes not even reported, and studies are limited to analyzing mainly systemic ones.

Only in the event of SRs is a deviation from the VIT administration protocol required in the build-up phase, with a dose decrease of two therapeutic steps and the resumption of the normal administration schedule from a previously tolerated dose, also taking into consideration premedication with an anti-H1 antihistamine [64]. In general, the literature on the subject reports how some risk factors, such as the use of rush and ultra-rush protocols, clonal mast cell pathologies have been detected as risk factors for SRs during VIT [1]. In any case, even if no fatal cases have been recorded in the literature to date, systemic reactions during VIT can be unpredictable, and the administration of this therapy must be by personnel with specific expertise in specialized centers with infrastructures ready to deal with any type of reaction [36]. For this reason, it is advisable, before each session, to investigate with the patient the effects of the last dose administered and any changes in health and to implement all necessary safety measures.

Theoretically, VIT can be suspended following negative skin tests and sIgE. However, in clinical practice, this occurs rarely, and only a small number of patients reach negative tests [82]. The recommended duration of VIT in children seems to be superimposable to what is suggested for adults. In the latest recommendations, its continuation is recommended for at least 5 years in the light of what emerged in a study [68]. In some particular cases, the possibility of continuing VIT beyond 5 years could be evaluated, for example, in the case in which there are specific risk factors for a new reaction after the interruption of therapy (Table 6).

**Table 6:** Risk factors of a new reaction after VIT interruption; modified from [1]

Hypersensitivity to honeybee venom (less protection than vespid venom)
Serious systemic reaction prior to initiation of specific immunotherapy
Systemic reactions triggered by the administration of specific immunotherapy
Systemic reactions triggered by a puncture during specific immunotherapy
Allergy tests (skin tests and/or serum specific sIgE) with persistence of a high degree of sensitization in the fifth year of specific immunotherapy
Clonal mast cell disease

In patients affected by clonal mast cell pathologies, lifelong VIT can even be recommended [1]. To date, there are no data on the long-term protection of VIT beyond 15 years.

During the follow-up of patients receiving immunotherapy, it is desirable to monitor sensitization by carrying out allergy tests (skin tests and sIgE) 3 and 5 years after the start of therapy and in each case of a new puncture. At the end of the VIT, however, it is advisable to carry out a control visit in each case of a new sting and at each appointment for the prescription of adrenaline together with the therapeutic education session [1]. After the administration of each single dose, to be performed with appropriate safety measures to exclude the onset of potential, although rare, severe adverse reactions, the patient must be observed for at least 1 h [36].

## Prevention

The prevention of Hymenoptera venom anaphylaxis in patients who have already developed a previous episode is crucial and must be supported by environmental protection interventions and early therapy. Places where one is more likely to encounter insects and risky behaviors should be avoided. By applying preventive actions and referring promptly to an allergist for correct diagnosis and adequate follow-up, it is possible to considerably reduce the risk of receiving a new Hymenoptera sting [83]. Moreover, an important step in susceptible subjects is the treatment of a new sting through the correct management [84] (Table 7).

**Table 7:** Actions to be taken in case of Hymenoptera sting in patients at risk for anaphylaxis

Own a plate or form with identification notes indicating the allergic condition
Get away as quickly - though cautiously - as possible from the scene of the accident
In the event of a honeybee sting, remove the sting immediately
Check the time immediately, as it is useful to evaluate the onset of clinical manifestations
If you are alone, try to reach an inhabited place or a first aid as soon as possible; report your condition and position to the emergency medical service at the initial clinical manifestations. If you are in company, immediately inform those around you and start the previous procedures together
In case of signs and symptoms appearance, take the necessary emergency medications, as personalized treatment plan

Detailed written information describing how to avoid any future sting should be provided and explained to patients allergic to Hymenoptera venom. An emergency medical kit must be supplied, which includes, e.g., antihistamines, steroids for local and oral use, and, most importantly, adrenaline auto-injectors, when needed, with a clear, practical demonstration under the supervision of a doctor or a qualified nurse [85]. Allergic patients must be informed of the possibility, when indicated, of undergoing VIT, the only treatment capable of modifying the natural history of the disease and improving their quality of life. The education of the patient on how to avoid future stings is a low-cost preventive measure that should be implemented. Based on the knowledge of the living conditions and habitat of the Hymenoptera, a series of recommendations have been formulated, which can potentially minimize the risk of a re-sting [84] (Table 8).

**Table 8:** Activities at risk for Hymenoptera stings and behaviors to adopt

**Examples of activities**	**Preventive actions**
**Eating and drinking outdoors**	Keep food covered and drinks closed until consumption; avoid cooking outdoors; keep the waste tightly closed, clean the edges of bins and spray them with insecticide
**Barefoot walking (especially on meadows and beaches)**	Wear closed shoes; avoid sudden movements if approached by a honeybee or a wasp
**Gardening (particularly cutting hedges or flowers)**	Wear gloves and pay attention to the use of electric mowers, which could inadvertently disturb or break hidden nests
**Fruit-picking; staying close to the hives when honey is collected**	Stay away from hedges, orchards, and vineyards; keep an insecticide in the car; beekeepers must employ protective measures (e.g., coveralls, masks, shoes)
**Outdoor sports**	Avoid brightly colored or black clothes, colognes, hair lotions and perfumed cosmetics, as they attract insects; do not wear shorts, low-cut dresses, unbuttoned shirts and clothes that can trap insects; try not to carry out recreational or work activities in the open air alone
**Removing vespid nests (from ceiling or windows)**	Provide the house windows with mosquito nets; entrust the cleaning of any honeybee hives or nests in the house or nearby to specialized personnel

Patients might be aware that Hymenoptera sting in self-defense and, therefore, all activities that are perceived by the animal as a potential danger may lead to a puncture. Individuals at risk should be provided with detailed information on the places where Hymenoptera build their nests (branches, log cavities, attics, chimneys, greenhouses, etc.), as well as foods that attract them (fruit, jam, honey, sweet drinks, etc.). Particular attention ought to be paid to the general measures of behavior of the pediatric age since information is reserved for the patients themselves, schoolteachers, and tutors of playful activities. In particular, it is necessary to exclude the carrying out of recreational activities in the open air alone. Finally, it should be considered that about 40%–85% of patients with fatal reactions to Hymenoptera stings do not have a history of previous anaphylaxis [4]. Therefore, it seems vital to achieve a correct awareness of the topic (with appropriate dissemination tools, e.g., social media and patient associations) and to improve knowledge of natural history and risk factors.

## Conclusion

This review elucidates Hymenoptera venom allergy in children, serving as a point of reference for clinicians caring for patients with this issue. However, the pediatric literature in the area is scarce, and data are often extrapolated from adult studies. Hence, collecting high-quality research data on children appears to be critical to establishing pediatric best practices in the field.

## Highlights

From a taxonomic point of view, Hymenoptera are subclassified into families: *Apidae*, including honeybees (*Apis mellifera*) and bumblebees (Bombus), and Vespidae, which, in turn, are divided into the subfamilies of Vespinae (wasps, including hornets, vespules, dolichovespules) and Polistinae (paper wasp). Hypersensitivity to Hymenoptera venom can be linked to immunological (IgE-mediated or non-IgE-mediated) and non-immunological mechanisms. Reactions are classified into local reactions, large local reactions, systemic reactions, toxic reactions, and unusual reactions. In general, children sensitize less frequently and have less severe reactions than adults, probably due to less exposure to repeated stings and fewer comorbidities. There are risk factors for systemic reactions that should be discussed with patients and their parents as appropriate. A correct diagnosis of Hymenoptera venom allergy relies on a careful clinical history and the appropriate use of skin and in vitro tests. The in vitro tests include serum specific IgE toward venom extracts and toward allergenic molecules. In complex diagnoses, CAP-inhibition and the Basophil Activation Test can also be used. In the presence of a systemic reaction, the basal serum tryptase measurement should be performed to rule out mastocytosis. In case of allergic reactions to Hymenoptera stings, in the acute phase, according to the current guidelines, the treatment of signs and symptoms mainly includes the use of adrenaline as first-line treatment in case of anaphylaxis and antihistamines and corticosteroids as subsequent lines of treatment. Given the impossibility of avoiding a new sting with certainty, the treatment of choice in subjects with hypersensitivity to Hymenoptera venom who have experienced systemic reactions is based on venom immunotherapy (VIT), with the venom of the responsible stinging insect identified after an adequate allergological work-up. VIT is performed in a suitable environment and has proved to be safe and effective with various administration protocols, both accelerated and conventional. The prevention of Hymenoptera venom anaphylaxis in patients who have already developed a previous episode is crucial and must be supported by environmental protection interventions and early therapy. Places where one is more likely to encounter insects and risky behaviors should be avoided.

## Data Availability

Not applicable.

## Abbreviations

BAT: Basophil Activation Test
EMA: European Medicines Agency
LLRs: Large local reactions
sIgE: Specific IgE
SPTs: Skin prick tests
SRs: Systemic reactions
VIT: Venom immunotherapy
